# The Antinociceptive Properties of the Corydalis *yanhusuo* Extract

**DOI:** 10.1371/journal.pone.0162875

**Published:** 2016-09-13

**Authors:** Lien Wang, Yan Zhang, Zhiwei Wang, Nian Gong, Tae Dong Kweon, Benjamin Vo, Chaoran Wang, Xiuli Zhang, Jae Yoon Chung, Amal Alachkar, Xinmiao Liang, David Z. Luo, Olivier Civelli

**Affiliations:** 1 Department of Pharmacology, University of California Irvine, Irvine, California, United States of America; 2 Department of Anesthesiology and Perioperative Care, University of California Irvine, Irvine, California, United States of America; 3 Dalian Institute of Chemical Physics, Chinese Academy of Sciences, Dalian, Liaoning, China; 4 Department of Pharmaceutical Sciences, University of California Irvine, Irvine, California, United States of America; 5 Developmental and Cell Biology, University of California Irvine, Irvine, California, United States of America; University of California Los Angeles, UNITED STATES

## Abstract

Corydalis *yanhusuo*. W.T. extracts (YHS) are widely used for the treatment of pain and inflammation. There are a few studies that assessed the effects of YHS in pain assays; however, none of these studies has systematically compared its activities in the different pain animal modes namely: acute, inflammatory and chronic pain. Furthermore, little is known about the mechanism of YHS activity in these assays. The aim of this study was to systematically evaluate the antinociceptive properties of YHS by testing it in four standardized pain assays and to investigate its mechanism. YHS antinociceptive properties were analyzed in the tail flick, the formalin paw licking, the von Frey filament and the hot box assays after spinal nerve ligation, which monitors acute nociceptive, persistent inflammatory and chronic neuropathic pain, respectively. YHS pharmacological profile was determined by screening it against a battery of G-protein coupled receptors and its mechanism of action was studied using knock-out mice. Our study shows that YHS, at a non-sedative dose, increases the tail flick latency in the tail flick assay without resulting in development of tolerance. YHS also decreases paw licking time in the formalin assay. Further, YHS increases paw withdraw threshold and latency in the von Frey filament and the hot box assays, respectively. *In vitro*, YHS exhibits prominent dopamine receptor antagonistic properties. In dopamine D2 receptor knockout mice, its antinociceptive effects are attenuated in acute and neuropathic pain but not inflammatory pain assays. Our results therefore indicate that YHS effectively attenuates acute, inflammatory and neuropathic pain, without causing tolerance. The effects on acute and neuropathic pain, but not inflammatory pain, are at least partially mediated through dopamine D2 receptor antagonism. Since YHS is a dietary supplement commercially available in the United States, our data suggest that it might be a candidate for alternative pain treatment.

## Introduction

Pain is an unpleasant sensory and emotional experience associated with tissue damage [1]. Pain management is currently limited, particularly for chronic pain. The potent opiate drugs are common and effective against some types of moderate to severe pain [2]. This class of drug, however, causes severe side effects [3, 4]. Anticonvulsants, antidepressants and selective serotonin noradrenaline reuptake inhibitors are used, but not exclusively, for neuropathic pain treatment with limited effectiveness [5]. Therefore, new analgesic alternatives are still being explored and pursued with every effort.

For centuries, varieties of extract from natural products, mostly plants, have been used for pain relief. Corydalis *yanhusuo*. W.T. Wang is officially listed in the Chinese Pharmacopoeia and its powder and decoction have been widely used for treatment of pain and inflammation [6]. However, only a few studies have investigated YHS antinociceptive properties in rodents assessed by standardized pain assays. None of the studies addressed concomitantly YHS effects in acute, inflammatory and neuropathic pain. Also these studies report large variations with respect to number of administrations (either single, 3 days or 5 days administration) and dosage (ranging from 30 mg/kg to 20 g/kg). Furthermore none of these studies addressed the mechanism of YHS effect [7–10]. YHS is known to contain several alkaloids [11–14]. However only l-tetrahydropalmatine (l-THP) and dehydrocorybulbine (DHCB) have been isolated and quantified in YHS. Both alkaloids display antinociceptive properties which rely at least partially, on dopamine D2 receptor antagonism [15, 16]. Therefore, we systematically evaluated the antinociceptive properties of YHS in three animal models of pain assays and investigated its pharmacological profile and mechanism of action.

## Materials and Methods

### Plant material and extraction

The tuber of Corydalis *yanhusuo* W.T. Wang was collected in Dongyang County (Zhejiang, China) and authenticated by Institute of Medication, Xiyuan Hospital of China Academy of Traditional Chinese Medicine. The tuber was first processed with vinegar as used in the traditional way to enhance the analgesic effect. The extraction of YHS was performed by Mai-Di Hai Pharmacy (China). The procedures were as follows: 10 kg of herb was ground into powder and decocted in 100 L of water at 100°C for 120 minutes. Then the residue was collected and redecocted in 100 L of water at 100°C for 90 minutes. The decoctions were pooled together and dried by spray drying to yield 0.6 kg water extract.

### Plasmid construction and stable cell lines

All G protein coupled receptors (GPCRs) used in this study were amplified from human cDNA library (Clontech, Palo Alto, CA) and cloned into pcDNA 3.1 (-) (Invitrogen, Carlsbad, CA). The sequences were confirmed by sequencing from both ends and with internal primers by Laragen (Los Angeles, CA). Human embryonic kidney-293 T cells (HEK293T) were cultured in Dulbecco’s Minimum Essential Medium (DMEM) supplemented with 10% fetal bovine serum (FBS). Serotonin 1A, 2A, 2C, adrenergic α1B, α2A, α2B, β1, β2 and neuokinin 1 receptors were transiently expressed in HEK293T cells. Briefly, plasmids were transfected into HEK293T cells with jetPRIME transfection reagents (Polyplus-transfection) following the manufacturer's recommendation. Three μg of each of the receptor and G protein chimera (either Gα15 or Gqαi3) were mixed in 750 ul of jetPRIME buffer and let stand at room temperature for 10 minutes after mixing with 12 μl of the transfection reagent. The mixture was added to cells without changing medium. The stable cell lines expressing dopamine D1, D2, muscarinic acetylcholine 1, histamine 2, melanin concentrating hormone 1, melatonin 1, opioid μ, δ and κ receptors were created individually. As an example, the stable cell lines expressing human opioid μ, δ or κ receptors were created as previously reported [17]. Briefly, the individual human opioid receptors μ, δ, or κ DNA plasmid were cotransfected with Gqαi3. Transfection was carried out with lipofectamine (Invitrogen, Carlsbad, CA) using the protocol provided by the supplier. Stable cell clones were selected in the presence of 200 μg/ml G418, 200 μg/ml hygromycin and 200 μg/ml zeocin.

### Fluorometric Imaging Plate Reader Assay (FLIPR)

The assay was performed as reported earlier [18]. Briefly, the stable cells were seeded into poly-D-lysine-coated black wall, clear-bottom 96-well plates at a density of 80,000 cells per well. Twenty-four hours later the medium was removed and replaced with 100 μl of dye loading solution (2 μM Fluo-4 AM dissolved in FLIPR buffer, which consists of pluronic acid in 1×Hank’s buffer supplemented with 20 mM HEPES, pH 7.4) for one hour at 37°C. The cells were then washed three times with FLIPR buffer prior to FLIPR assay. The samples, which were re-dissolved in dimethyl sulphoxide (DMSO) and stored in 96-well drug plates, were diluted with FLIPR buffer and then added into the cells within 4s automatically. For agonist tests, the intracellular Ca^2+^ concentration was monitored at 520 nm with excitation wavelength at 488 nm over a period of 4 minutes. For antagonist tests, the compound was first incubated with the cell for 10 minutes, before the addition of intrinsic receptor ligand with EC50 determined in individual receptor expressing cell lines. Data were expressed as fluorescence (arbitrary units) versus time.

### Animals

Male Swiss Webster mice (age 8–12 weeks, Charles River, Wilmington, MA) were used in the tail flick, formalin, locomotion and rotarod assays. Male 129/sv mice (age 8–12 weeks, Charles River, Wilmington, MA) were used in the von Frey filament, hot box and locomotion assays. Male dopamine D2 receptor knockout mice (age 8–12 weeks) were used for mechanistic studies (tail flick, formalin, von Frey filament, hot box, locomotion and rotarod assays). Age-matched wild-type littermates with the same genetic background were used as control animals. The generation of dopamine D2 knockout mice was reported previously [19]. Mice were group-housed and maintained on a 12-hours light/dark cycle (light on at 7:00am) with food and water available ad libitum. All experimental procedures were approved by the Institutional Animal Care and Use Committee of University of California, Irvine and were performed in compliance with national and institutional guidelines for the care and use of laboratory animals. Apart from the daily monitoring of common animal health, animals that were subjected to the tail flick and hot box assays were monitored for potential tissue injury. Animals with tissue injury after the assays were excluded from further assessment. Animals that were subjected to the formalin paw assay were euthanized with CO_2_ overdose followed by cervical dislocation immediately after the assessment to alleviate inflammation induced distress.

### Drug Administrations

YHS was dissolved in a vehicle solution of cremophor EL: ethanol: saline (2:1:17). Morphine sulphate (Sigma-Aldrich) was dissolved in saline. Dehydrocorybulbine (DHCB) was synthesized as previously reported [16] and dissolved in saline. L-tetrahydropalmatine (l-THP, Xi,an Xiaocao Botianical Development Co.,LTD) was dissolved in saline. YHS (100, 200, 250, 500 mg/kg, i.p., 5 ml/kg) and morphine (10 mg/kg, i.p., 5 ml/kg) were administered at different time points depending on the assays described in detail below. We also compared YHS efficacy to that of a mixture of l-THP and DHCB. YHS contains approximately 0.25% of l-THP and 0.18% of DHCB [16]. Therefore, a 500 mg/kg YHS administration (i.p., 5 ml/kg) was compared to the administration of a mixture of 1mg/kg l-THP (i.p., 5 ml/kg) and 1mg/kg DHCB (i.p., 5 ml/kg) in the tail flick assay. Both Solutions were administered 30 minutes before the assay.

### Behavioral Testing

#### Tail-flick assay

The tail-flick assay was performed as described before [16]. Briefly, the tail removal latency was measured using an electronically controlled tail-flick analgesimeter (UGO basile biological research apparatus, 7360 Tail Flick) that integrated both a thermal nociceptive stimulus and an automated response timer. Mice were applied with a thermal stimulus (focused light from a 20W infrared bulb as the heat source) directed to the tips of their tails. The time from onset of stimulation to a rapid withdrawal of their tails from the heat source was recorded as tail flick latency. These experiments were carried out using a moderate 6–7 seconds of baseline to permit low antinociception detection. A maximum of 22 seconds was set as a cut off time to prevent tissue damage. After three consecutive daily baseline measurements, mice were administered with vehicle, morphine (10 mg/kg), YHS (100–500 mg/kg) or the mixture of DHCB (1 mg/kg) and l-THP (1 mg/kg) and tail flick latency was measured immediately before the injections and 30, 60, 120 and 180 minutes after the injection.

#### Formalin paw assay

The formalin paw assay was performed as described before [20]. Briefly, mice were placed individually in a 4 liter glass beaker and were allowed to acclimate for 30 minutes before the test. Vehicle, morphine (10 mg/kg), or YHS (200 mg/kg) were administered 15 minutes prior to formalin injection. Twenty five μl of 0.5% formalin solution was administered into the dorsal surface of the right hind paw using a 50 μl Hamilton syringe with a 30 gauge needle. Immediately after formalin injection, mice were placed individually in the beaker and a mirror was arranged at a 45° angle under the beaker to allow clear observation of the paws. The nociceptive behavior (paw licking) was observed continuously for 50 minutes. The time animals spent licking the injected paw during the first phase (0–10 minutes) and second phase (10–50 minutes) were recorded in a 5-minute intervals.

#### Spinal nerve ligation (SNL) surgery

Unilateral SNL injury was performed as described before [21]. Briefly, the left L4 spinal nerve [22] was exposed in an isoflurane anesthetized mouse and ligated with a silk suture between dorsal root ganglia and the conjunction of sciatic nerve. Sham operations were performed in the same way except that spinal nerves were not ligated. The drug administration was carried out about two weeks post SNL when all experimental mice have developed hindpaw mechanical and thermal hypersensitivities on the injured side, assessed by the von Frey filaments and hot box assay, respectively.

#### Von Frey filaments assay

Paw withdrawal thresholds (PWT) to calibrated von Frey filament (Stoelting, Wood Dale, IL) stimulation were assessed in both hindpaws with a modified up-down method of Dixon [23] before and after SNL as described previously [24]. Briefly, mice were acclimated for 60 minutes on a mesh surface of the test apparatus. Mice were then administered vehicle or YHS (200 mg/kg). A series of von Frey filaments (buckling force between 0.04 and 2.0 g), starting with 0.4 g, was applied to the hindpaw plantar surface before the injection and 60, 120 and 240 minutes after the injection. A positive response of paw lifting within 5 seconds led to the use of the next weaker filament. Absence of paw lifting after 5 seconds was considered a negative response and led to the use of the next filament with increasing force. Scores of six measurements, starting from the one prior to the first positive response, were used to calculate the 50% PWT except that a score of 0.01 g was assigned to four consecutive positive responses or a score of 3.0 g was assigned to three consecutive negative responses.

#### Hargreaves assay

Mouse hindpaw withdrawal latencies (PWL) from a thermal stimulus were measured in a modified Hargreaves-type hot box as described before [25]. Briefly, mice were acclimated for at least 60 minutes within individual boxes on the hot box glass surface maintained at 30°C (developed by University of California, San Diego). Mice were then administered vehicle or YHS (200 mg/kg). A radiant light source under the glass surface was aligned to the hindpaw plantar surface before the injection and 60, 120 and 240 minutes after the injection. A timer was activated when the light source was turned on and stopped when a paw withdrawal from the light source was detected by motion sensors or at 20 seconds of light stimulation to prevent thermal injury.

#### Tolerance assay

The tolerance study was performed using a repeated-injection schedule as described before [26]. Mice were administered with vehicle, morphine (10 mg/kg), or YHS (200 mg/kg) once daily for consecutive 7 days. The loss of the antinociceptive effects of drugs in the tail-flick test was used to assess the degree of tolerance. The procedure of the tail flick assay was described above in the “Tail-flick assay” section. The tail-flick latency was assessed on the 1^st^, 3^rd^, 5^th^ and 7^th^ day, 30 minutes after the injection.

#### Spontaneous locomotor activity assay

The sedative effects of the drugs on the animals were evaluated by measuring the spontaneous locomotor activity for 60 minutes as previously reported [27]. The habituation step was skipped because of the possible sedative effect of the test drugs. Thirty minutes after mice were administered with vehicle or YHS (100–500 mg/kg), they were placed into an open field test chamber (40 x 40 cm, Med Associates, inc.). Horizontal movements were measured by the infrared beams arrays. The total distance animals travelled for 60 minutes was recorded, analysized and calculated by Activity Monitor 5 software (Med Associates, inc.); it is used to evaluate the effects of drugs on locomotion.

#### Rotarod assay

The rotarod assay was performed as described before with modifications [28]. Mice were trained to remain on the rotarod apparatus (TSE Systems, Inc.) before the test session for two consecutive days, two trials per day (9:00 am and 3:00 pm). The first training trial consisted of a 5 minute interval followed by an accelerating speed ranging from 4–20 rotations per minute (rpm) for 180 seconds and then at constant speed at 20 rpm for 120 seconds. The other three trials consisted of only constant speed at 20 rpm for 120 seconds. Animals which could easily stay on the rod for at least 60 seconds were used. On the third day, mice were placed on the rotarod 60 minutes after treatment with vehicle or YHS (100–500 mg/kg), and the latency to fall off the rotarod within 120 seconds was recorded automatically by an infrared beam located below the rotating rod.

### Data analysis

Graphpad Prism (GraphPad Software, Inc.) was used for statistical analysis. Data are presented as means ± S.E.M. Results were analyzed by student’s t test or ANOVA followed by the appropriate post hoc comparisons, and *P* < 0.05 was considered statistically significant.

## Results

### The antinoceceptive properties of YHS

**Fig 1:**
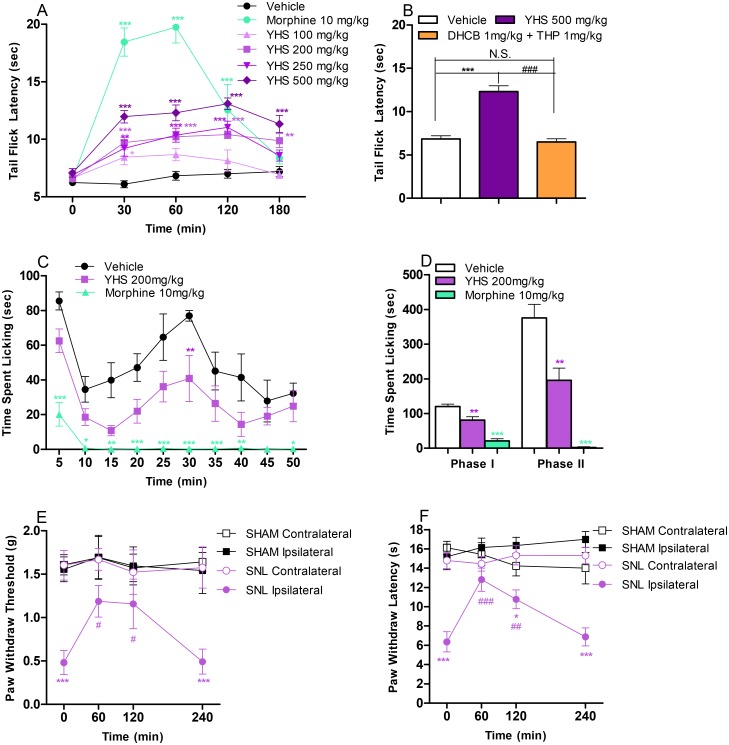
Antinociceptive effects of YHS in acute, inflammatory and neuropathic pain models. (A) Antinociceptive effects of YHS (100–500 mg/kg) in the tail flick assay (n = 7–9). Two way ANOVA revealed significant drug effects (F_6,49_ = 38.26, P < 0.0001), time effect (F_4,196_ = 59.93, P < 0.0001) and drug x time interaction (F_24,196_ = 14.15, P < 0.0001) followed by Bonferroni post hoc test: drug vs vehicle, *P < 0.05, **P < 0.01, *** *P* < 0.001. (B) Comparison between YHS (500 mg/kg) and the mixture of l-THP (1 mg/kg) and DHCB (1 mg/kg) in the tail flick assay (n = 8–9). Tail-flick latencies were measured 60 min following drug administration. One way ANOVA revealed a significant drug effect (F_2,22_ = 42.85, P < 0.0001) followed by Bonferroni post hoc test: drug vs vehicle, *** *P* < 0.001, N.S., not significant; YHS vs L-THP+DHCB, ^###^ *P* < 0.001. (C) Time course of the antinociceptive effects of YHS (200 mg/kg) in the formalin paw licking assay (n = 6–7). Two way ANOVA revealed significant drug effects (F_2,17_ = 36.59, P < 0.0001) and time effect (F_9,153_ = 10.22, P < 0.0001) followed by Bonferroni post hoc test: drug vs vehicle, *P < 0.05, **P < 0.01, *** *P* < 0.001. (D) Cumulative effects of the antinociceptive effects of YHS (200 mg/kg) in the formalin paw licking assay (n = 6–7). One-way ANOVA indicated significant drug effects in both Phase I (F_2,17_ = 34.92, P < 0.0001) and Phase II (F_2,17_ = 31.68, P < 0.0001) followed by Dunnett’s post hoc tests: drug vs vehicle, **P < 0.01, *** *P* < 0.001. (E) Antinociceptive effects of YHS (200 mg/kg) in the von Frey filament assay after spinal nerve ligation (n = 10). Two way ANOVA revealed a significant treatment effect (F_3,36_ = 11.42, P < 0.0001) followed by Bonferroni post hoc test: contralateral vs. ipsilateral, *** *P* < 0.001. (F) Antinociceptive effects of YHS (200 mg/kg) in the hot box assay after spinal nerve ligation (n = 10). Two way ANOVA revealed a significant treatment effect (F_3,36_ = 31.06, P < 0.0001) and drug x time interaction (F_9,108_ = 3.045, P = 0.0028) followed by Bonferroni post hoc test: *P < 0.05, *** *P* < 0.001.

The antinociceptive properties of YHS were first tested in the tail-flick assay, which records responses to an acute thermal stimulus. The results show that YHS significantly increased the tail flick latencies in a dose dependent manner for a minimum of 3 hours (*P* < 0.05, Fig 1A). Noteworthy, two active components of YHS, l-THP and DHCB exhibited antinociceptive effects with a minimum dose of 10 mg/kg [16]. As mentioned in the method section, 500 mg of YHS contains approximately 1 mg of l-THP and 1 mg of DHCB. We show that YHS at 500 mg/kg displays a significant antinociceptive effect (*P* < 0.001, Fig 1B), while a mixture of l-THP and DHCB at 1 mg/kg had no effect (*P* > 0.05, Fig 1B), possibly indicating that other components in YHS are necessary for full antinociceptive activity. Most analgesics possess sedative properties, and strong sedation may mask the assessment of pain. Thus, YHS was tested for its potential sedative properties by using locomotor activity and rotarod assays. The results show that up to 200 mg/kg YHS did not significantly reduce locomotor activity nor affect rotarod (*P* > 0.05, S1A and S1B Fig). We consequently used 200 mg/kg as the nonsedative effective dose in the subsequent studies.

We extended our study to evaluate the antinociceptive properties of YHS in the formalin paw assay, which measures both acute and chronic inflammatory responses [29]. As shown in Fig 1C and 1D, YHS at 200 mg/kg significantly reduces the time mice spent licking the paw in both the early phase which corresponds to acute neurogenic pain (*P* < 0.01), and the late phase which corresponds to inflammatory pain (*P* < 0.01). YHS was further tested on animals after SNL in the von Frey filament and hot box assays, which measure mechanical allodynia and thermal hyperalgesia respectively. We show that YHS at a non-sedative dose (200 mg/kg, determined in S1C Fig) significantly increases PWT and PWL in the von Frey filament and hot box assays, respectively, for a minimum of 2 hours (P > 0.05, Fig 1E and 1F). Mice treated with vehicle show no effect on either assays (data not shown). Taken together, these data demonstrate that at a non-sedative dose, YHS is effective in suppressing nociceptive responses to thermally induced acute pain, chemically induced inflammatory pain as well as injury induced neuropathic pain.

### The mechanism of YHS antinociceptive activity

We then investigated the potential mechanism of YHS antinociceptive effects. YHS was first screened against a battery of GPCRs using the FLIPR assay. As summarized in Table 1, YHS exhibits antagonistic properties mostly to dopamine D1 and D2 receptors (dose response curves shown in S2 Fig). The Dopamine D2 receptor has been proposed to play an important role in pain and analgesia [30]. Besides, l-THP and DHCB have been previously shown to exert their antinociceptive effects partially through D2 receptor antagonism [15, 16]. Therefore, we tested whether dopamine D2 receptor is involved in YHS antinociception by using dopamine D2 receptor knockout (D2KO) mice.

**Table 1:** Pharmacological profiles of YHS within G protein coupled receptors (GPCRs).

CNS Target	YHS Activity
Dopamine D1	3.129 ± 0.2423 (Antagonism)
Dopamine D2	3.091 ± 0.1893 (Antagonism)
Serotonin 1A/2A/2C/6	None
Adrenergic α1B/α2A/α2B/β1/β2	None
Muscarinic Acetylcholine R1	None
Histamine R2	None
Melatonin R1	None
Opioid μ, δ, κ	None
Opioid receptor-like R	None
Melanin concentrating hormone R1	None
Neurokinin R1	None

IC50 (μg/ml) values are presented as mean±S.E.M.

**Fig 2:**
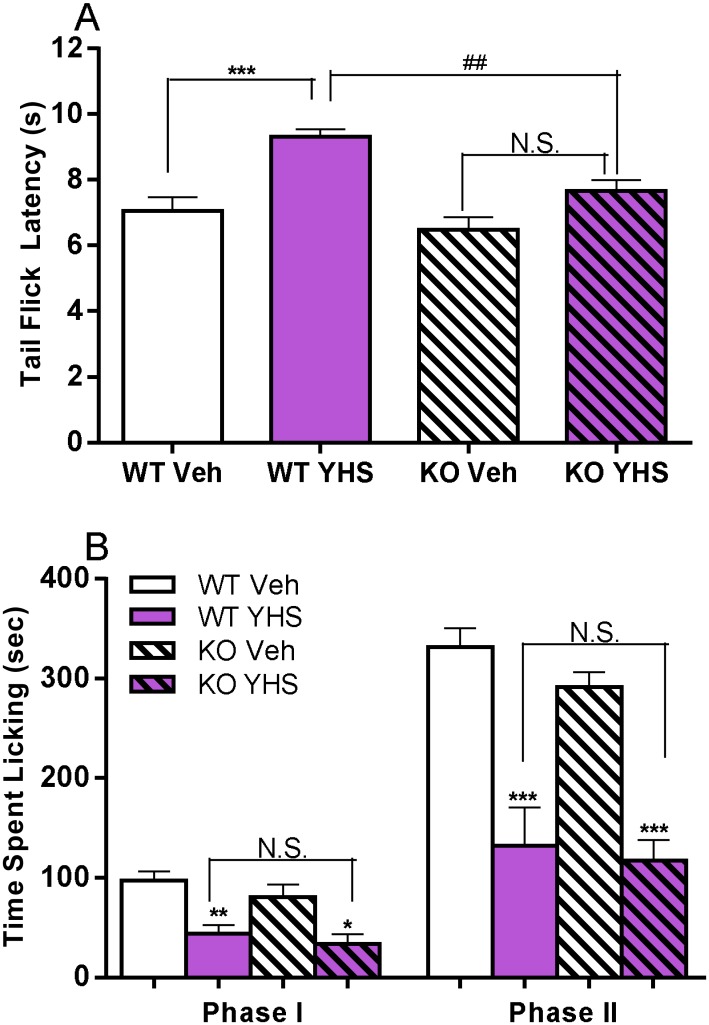
Effects of YHS in D2KO mice assessed in the tail flick and formalin paw licking assays. (A) Effects of YHS (200 mg/kg) in D2KO mice assessed in the tail flick assay (n = 10). Tail-flick latencies were measured 120 min following drug administration. One-way ANOVA indicated a significant drug effect (F_3,36_ = 12.53, P < 0.0001) followed by Bonferroni post hoc test: YHS vs vehicle, *** *P* < 0.001, N.S., not significant; KO vs WT, ^##^ *P* < 0.01. (B) Effects of YHS (200 mg/kg) in D2KO mice assessed in the formalin paw licking assay (n = 6). One-way ANOVA indicated significant drug effects in both Phase I (F_3,20_ = 8.751, P = 0.0007) and Phase II (F_3,20_ = 19.01, P < 0.0001) followed by Bonferroni post hoc test: YHS vs vehicle, *P < 0.05, **P < 0.01, *** *P* < 0.001; KO vs WT, N.S., not significant.

In the tail flick assay, YHS at a non-sedative dose (200 mg/kg, determined in S1D and S1E Fig), induces antinociceptive effect in wild type (WT, *P* < 0.001, Fig 2A) but not in D2KO mice (*P* > 0.05, Fig 2A). In the formalin assay, however, YHS at 200 mg/kg induces a similar level of antinociceptive responses in both WT and D2KO mice (*P* < 0.05, Fig 2B) with no significant difference (*P* > 0.05, Fig 2B). These results indicate that the antinociceptive effect of YHS in acute pain but not inflammatory pain is attenuated in D2KO mice.

**Fig 3:**
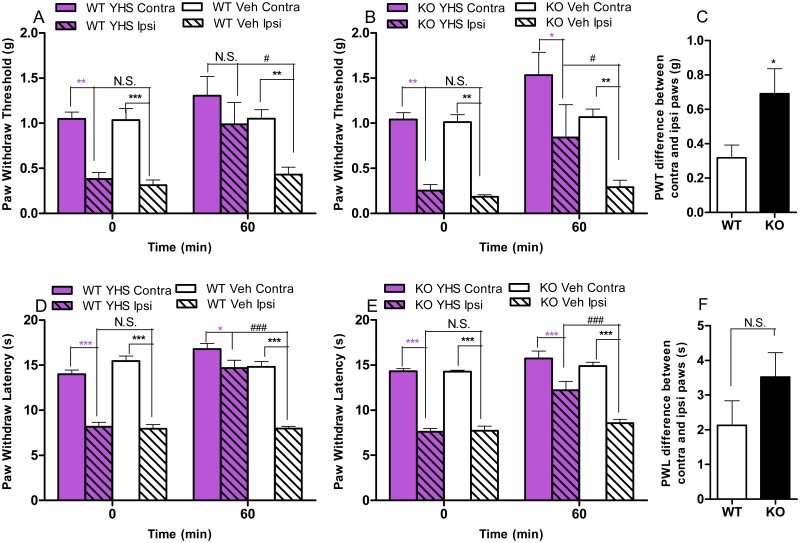
Effects of YHS in D2KO mice assessed in the von Frey filaments and hot box assays. (A) Effects of YHS (200 mg/kg) in WT mice assessed in the von Frey filaments assay after SNL (n = 10). Paw withdraw thresholds were measured 60 minutes after drug administration. Two way ANOVA revealed a significant treatment effect (F_3,36_ = 12.81, P < 0.0001) and time effect (F_1,36_ = 7.244, P = 0.0107) followed by Bonferroni post hoc test: contralateral vs. ipsilateral, **P < 0.01, *** *P* < 0.001, N.S., not significant; YHS vs vehicle, ^#^ *P* < 0.05. (B) Effects of YHS (200 mg/kg) in D2KO mice assessed in the von Frey filaments assay after SNL (n = 10). Paw withdraw thresholds were measured 60 minutes after drug administration. Two way ANOVA revealed a significant treatment effect (F_3,36_ = 17.00, P < 0.0001) and time effect (F_1,36_ = 6.340, P = 0.0164) followed by Bonferroni post hoc test: contralateral vs. ipsilateral, *P < 0.05, **P < 0.01; YHS vs vehicle, ^#^ *P* < 0.05, N.S., not significant. (C) Paw withdraw threshold (PWT) difference between contralateral (Contra) and ipsilateral (Ipsi) paws in WT and D2KO mice assessed in the von Frey filaments assay 60 minutes after YHS (200 mg/kg) administration (n = 10): t = 2.281, *P* = 0.0349. Unpaired student t test, KO vs WT, *P < 0.05. (D) Effects of YHS (200 mg/kg) in WT mice assessed in the hot box assay after SNL (n = 10). Paw withdraw latencies were measured 60 minutes after drug administration. Two way ANOVA revealed a significant treatment effect (F_3,36_ = 66.27, P < 0.0001), time effect (F_1,36_ = 34.40, P < 0.0001) and drug x time interaction (F_3,36_ = 19.38, P < 0.0001) followed by Bonferroni post hoc test: contralateral vs. ipsilateral, *P < 0.05, *** *P* < 0.001; YHS vs vehicle, ^###^ *P* < 0.001, N.S., not significant. (E) Effects of YHS (200 mg/kg) in D2KO mice assessed in the hot box assay after SNL (n = 9–10). Paw withdraw latencies were measured 60 minutes after drug administration. Two way ANOVA revealed a significant treatment effect (F_3,34_ = 75.99, P < 0.0001), time effect (F_1,34_ = 25.25, P < 0.0001) and drug x time interaction (F_3,34_ = 6.119, P = 0.0019) followed by Bonferroni post hoc test: contralateral vs. ipsilateral, *** *P* < 0.001; YHS vs vehicle, ^###^ *P* < 0.001, N.S., not significant. (F) Paw withdraw latency (PWL) difference between contralateral (Contra) and ipsilateral (Ipsi) paws in WT and D2KO mice assessed in the hot box assay 60 minutes after YHS (200 mg/kg) administration (n = 9–10): t = 1.394, *P* = 0.1812. Unpaired student t test, N.S., not significant.

In the von Frey filament assay (after SNL), YHS at 200 mg/kg shows antinociceptive effect (compared between contralateral and ipsilateral paws) in WT (*P* > 0.05, Fig 3A) but not in D2KO mice (*P* < 0.05, Fig 3B). Compared to WT mice, D2KO mice treated with YHS show a significant increase of the PWT difference between contralateral and ipsilateral paws (*P* < 0.05, Fig 3C, derived from the difference between the contralateral and ipsilateral paws in terms of PWT and PWL values within each genotype, respectively), which indicates an attenuated antinociceptive effect of YHS in D2KO mice. In the hot box assay, YHS at 200 mg/kg induces antinociceptive effects (compared between contralateral and ipsilateral paws) in both WT (*P* < 0.05, Fig 3D) and D2KO mice (*P* < 0.001, Fig 3E). Compared to WT mice, D2KO mice treated with YHS show an increase of the PWL difference between contralateral and ipsilateral paws, though not statistical significant (*P* > 0.05, Fig 3F, derived from the difference between the contralateral and ipsilateral paws in terms of PWT and PWL values within each genotype, respectively).

Taken together, these data indicate that the antinociceptive effects of YHS are mediated, at least partially, through dopamine D2 receptor in acute and neuropathic pain, but not in inflammatory pain.

### Lack of antinociceptive tolerance of YHS extract

**Fig 4:**
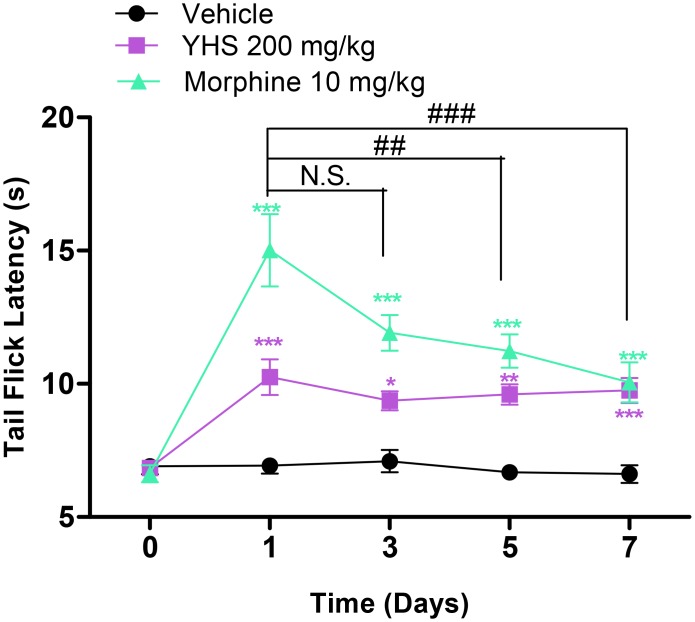
Lack of antinociceptive tolerance of YHS (200 mg/kg) in the tail flick assay (n = 7–8). Two way ANOVA revealed a significant drug effect (F_2,19_ = 68.24, P < 0.0001), time effect (F_4,76_ = 19.16, P < 0.0001) and drug x time interaction (F_8,76_ = 7.509, P < 0.0001) followed by Bonferroni post hoc test: drug vs vehicle, *P < 0.05, **P < 0.01, *** *P* < 0.001. One way ANOVA revealed a significant time effect in morphine treated group (F_3,24_ = 5.520, P = 0.005) but not in YHS treated group (F_3,28_ = 0.6032, P = 0.6184) followed by Dunnett’s post hoc tests: Day 3,5,or 7 vs Day 1, ^##^ *P* < 0.01, ^###^ *P* < 0.001, N.S., not significant.

Because one of the major drawbacks of the narcotic analgesics is the development of tolerance, we tested YHS for the development of tolerance to its antinociceptive effect. We show that unlike morphine (*P* < 0.01, Fig 4) YHS does not result in development of tolerance after daily administration for seven consecutive days (*P* > 0.05, Fig 4).

## Discussion

Pain can be classified into different types: nociceptive pain activated by noxious physical stimulus, inflammatory pain activated by the immune system upon tissue injury and neuropathic pain caused by damage to the nervous system resulting from physical damage or disease affecting the somatosensory system [1]. Our study is the first to systematically evaluate the antinociceptive properties of YHS in three types of pain assays (Fig 1). We show that YHS does not result in development of antinociceptive tolerance after 7 days of once-daily administration (Fig 4). We further characterized the pharmacological profile of YHS (Table 1) and demonstrated that antinociceptive effects of YHS are partially attributed to the interaction with dopamine D2 receptor in acute and neuropathic pain, but not inflammatory pain (Figs 2 and 3).

Our study confirmed the antinociceptive effects of YHS in the tail flick and the formalin paw licking assays, but also in the von Frey filament and the hot box assays after spinal nerve ligation, which monitor acute, inflammatory and neuropathic pain sensations, respectively. Besides, we show that YHS exhibits more antinociceptive efficacy than its two active components l-THP and DHCB. This suggests that YHS contains other active components which combine to produce its analgesic activity. Indeed several alkaloids other than l-THP and DHCB have been identified in YHS [11–14]. Among these, berberine, palmatine, columbamine and glaucine, identified in plant extracts others than YHS, have been reported to display antinociceptive properties in rodents [31–35]. These studies support our suggestion that the additive or synergistic effects of all these alkaloids, and also other unidentified active components, give YHS its advantageous antinociceptive property. Moreover, we show that repeated YHS administration does not lead to development of tolerance and therefore YHS may present advantages over morphine in chronic pain treatment.

To better understand the mechanism of the antinociceptive effects of YHS, we screened YHS against a number of GPCRs. Herb extracts, which contain varieties of compounds, often exhibit multiple receptor activities. In our screening which covered 20 receptors, YHS exhibited activities only at dopamine D1 and D2 receptors and these were antagonistic activities. This is in line with the antagonistic activities of its most studied components l-THP and DHCB [15, 16]. Based on these results, we investigated YHS activity on dopamine receptors *in vivo*.

Our study shows that in D2KO mice, the antinociceptive effects of YHS are significantly decreased in acute and neuropathic pain assays. These results indicate that its actions are partially mediated through the blockade of the dopamine D2 receptor. A central role of dopaminergic transmission in modulating pain perception within supraspinal and spinal regions has been demonstrated [30]. Striatal or spinal cord level of dopamine D2 receptor stimulation has been shown to exhibit antinociceptive effects in acute pain [36–38] and neuropathic pain [39–42]. These seemingly contradictory results could be explained by the preferential presynaptic D2 autoreceptor activation of some of the D2 receptor antagonists. It has been reported that a variety of D2 receptor antagonists at doses lower than that required for antipsychotic effects results in an increase in dopamine metabolism [43]. Low doses of amisulpride, a selective antagonist of the D2/D3 receptor with selective preference for presynaptic autoreceptors, show antinociceptive effects in acute pain assays [44]. Moreover, l-THP and DHCB have also been shown to exert their antinociceptive effects, at least partially, through D2 receptor antagonism [15, 16]. Additional studies are needed to determine if the antinociceptive effects of YHS in acute and neuropathic pain are regulated by presynaptic D2 receptors. Studies have shown that both D1 and D2 receptors are involved in mediating inflammatory pain responses in rodents [45–47]. Our study shows that the antinociceptive effect of YHS is not affected in D2KO mice. In this respect, whether or not the D1 receptor mediates YHS effects in inflammatory pain needs further investigation.

Consistent with our previous study [16], the D2KO mice display similar tail flick latency baseline as compared to WT mice in the tail flick assay (data not shown). WT and D2KO mice also display similar baseline in time spent licking in the formalin assay (Fig 2B). Our study is the first to characterize the neuropathic pain development after SNL in D2KO mice. It has been reported that under baseline conditions, D2KO mice show slightly longer PWL in the hot box assay. These mice are, however, slightly more sensitive to mechanical stimulation in the von Frey filament assay, and they do not show any difference in thermal and visceral pain responses [48]. In our study, under baseline conditions, WT and D2KO animals display similar overall PWT (Before SNL point in S3A Fig) in the von Frey filament assay. However, after SNL, D2KO mice develop a faster mechanical allodynia (Day 1 and 3 post SNL points in S3A Fig). Consequently both genotypes reached a similar level of mechanical sensitivity (Day 7 post SNL points in S3A Fig). On the other hand, WT and D2KO animals display similar overall PWL baseline (Before SNL point in S3B Fig) and a comparable trend in terms of thermal hyperalgesia development (Day 4 and 8 post SNL points in S3B Fig) in the hot box assay.

In summary, our study demonstrated the effectiveness of Corydalis *yanhusuo* extract in three types of pain assays without resulting in development of tolerance. Our study also illustrated the mechanism involved in these antinociceptive effects. It is noteworthy that YHS is not only clinically used for pain management in China, but also is sold as a dietary supplement in United States. Our current results suggest that YHS might serve as a candidate for alternative management of pain.

## Supporting Information

S1 Fig Effects of YHS in locomotor activity and rotarod assays. (A) Effects of YHS (100–500 mg/kg) in Swiss Webster mice assessed in the locomotor activity assay (n = 6–9). One way ANOVA revealed a significant drug effect (F_4,31_ = 13.66, P < 0.0001) followed by Dunnett’s post hoc tests: drug vs vehicle, **P < 0.01, *** *P* < 0.001. (B) Effects of YHS (100–500 mg/kg) in Swiss Webster mice assessed in the rotarod assay (n = 8). One way ANOVA revealed no significant drug effect (F_3,28_ = 0.7925, P = 0.5083). (C) Effects of YHS (100, 200 mg/kg) in 129/sv mice assessed in the locomotor activity assay (n = 9–10). One way ANOVA revealed no significant drug effect (F_2,25_ = 0.7993, P = 0.4608). (D) Effects of YHS (100, 200 mg/kg) in the mice used as wild-type control for the D2KO mice assessed in the locomotor activity assay (n = 8–10). One way ANOVA revealed no significant drug effect (F_2,24_ = 1.167, P = 0.3284). (E) Effects of YHS (100, 200 mg/kg) in the mice used as wild-type control for the D2KO mice assessed in the rotarod assay (n = 8). One way ANOVA revealed no significant drug effect (F_3,21_ = 0.5191, P = 0.6025)
(TIF)

S2 Fig The antagonist dose-response curve of YHS induced by dopamine in D1 and D2 receptors in D1 and D2 expressing cells. Error bars represent standard error of the mean of duplicate measurements for each point.
(TIF)

S3 Fig Neuropathic pain development of WT and D2KO mice. (A) Development of tactile allodynia of WT and D2KO mice assessed in the von Frey filaments assay (n = 10). Two way ANOVA revealed significant treatment effects (F_3,36_ = 11.99, P < 0.0001), time effect (F_3,108_ = 8.557, P < 0.0001) and treatment x time interaction (F_9,108_ = 2.451, P = 0.0140) followed by Bonferroni post hoc test: contralateral vs ipsilateral, *P < 0.05, *** *P* < 0.001. (B) Development of thermal hyperalgeisa of WT and D2KO mice assessed in the hot box assay (n = 10). Two way ANOVA revealed significant treatment effects (F_3,36_ = 56.34, P < 0.0001), time effect (F_2,72_ = 84.75, P < 0.0001) and treatment x time interaction (F_6,72_ = 16.90, P < 0.0001) followed by Bonferroni post hoc test: contralateral vs ipsilateral, *** *P* < 0.001.
(TIF)
