# Supplementary figures and images for: The Antinociceptive Properties of the Corydalis yanhusuo Extract
Source: PLoS One. 2016 Sep 13;11(9):e0162875. doi: 10.1371/journal.pone.0162875 (PMC5021270; doi:10.1371/journal.pone.0162875)

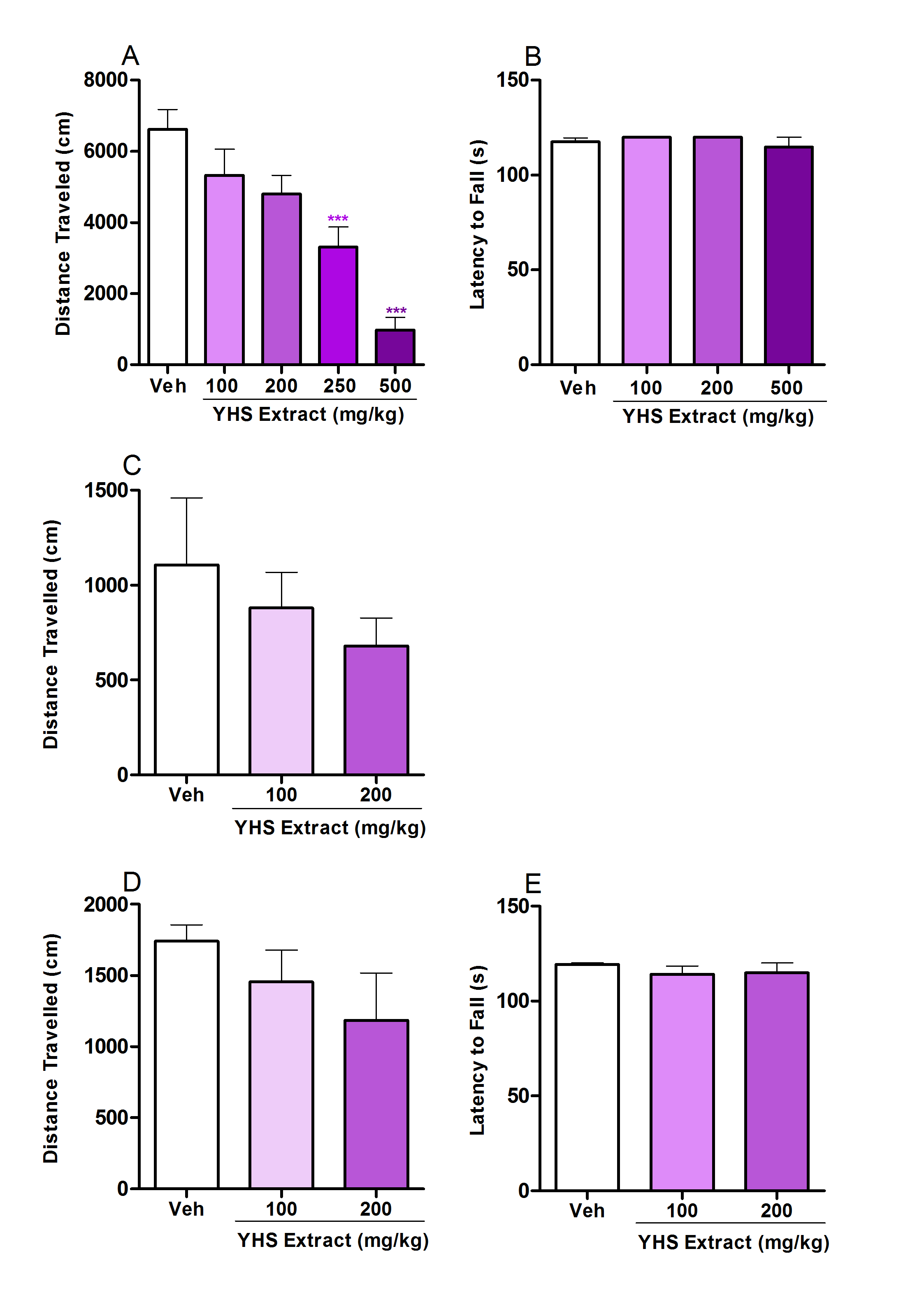

Supplement: S1 Fig — (A) Effects of YHS (100–500 mg/kg) in Swiss Webster mice assessed in the locomotor activity assay (n = 6–9). One way ANOVA revealed a significant drug effect (F4,31 = 13.66, P < 0.0001) followed by Dunnett’s post hoc tests: drug vs vehicle, **P < 0.01, *** P < 0.001. (B) Effects of YHS (100–500 mg/kg) in Swiss Webster mice assessed in the rotarod assay (n = 8). One way ANOVA revealed no significant drug effect (F3,28 = 0.7925, P = 0.5083). (C) Effects of YHS (100, 200 mg/kg) in 129/sv mice assessed in the locomotor activity assay (n = 9–10). One way ANOVA revealed no significant drug effect (F2,25 = 0.7993, P = 0.4608). (D) Effects of YHS (100, 200 mg/kg) in the mice used as wild-type control for the D2KO mice assessed in the locomotor activity assay (n = 8–10). One way ANOVA revealed no significant drug effect (F2,24 = 1.167, P = 0.3284). (E) Effects of YHS (100, 200 mg/kg) in the mice used as wild-type control for the D2KO mice assessed in the rotarod assay (n = 8). One way ANOVA revealed no significant drug effect (F3,21 = 0.5191, P = 0.6025) (TIF) [file pone.0162875.s001.tif]

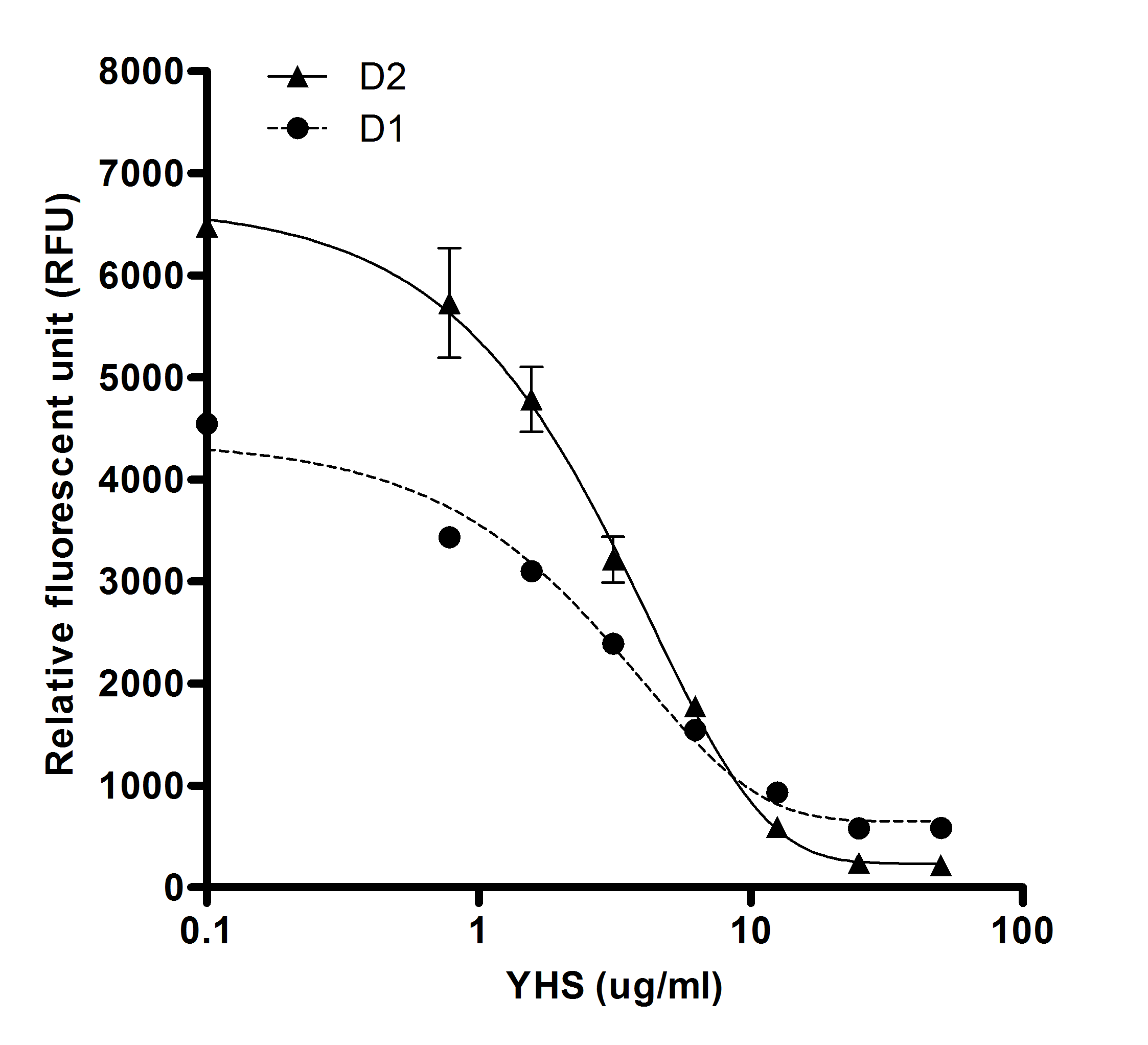

Supplement: S2 Fig — Error bars represent standard error of the mean of duplicate measurements for each point. (TIF) [file pone.0162875.s002.tif]

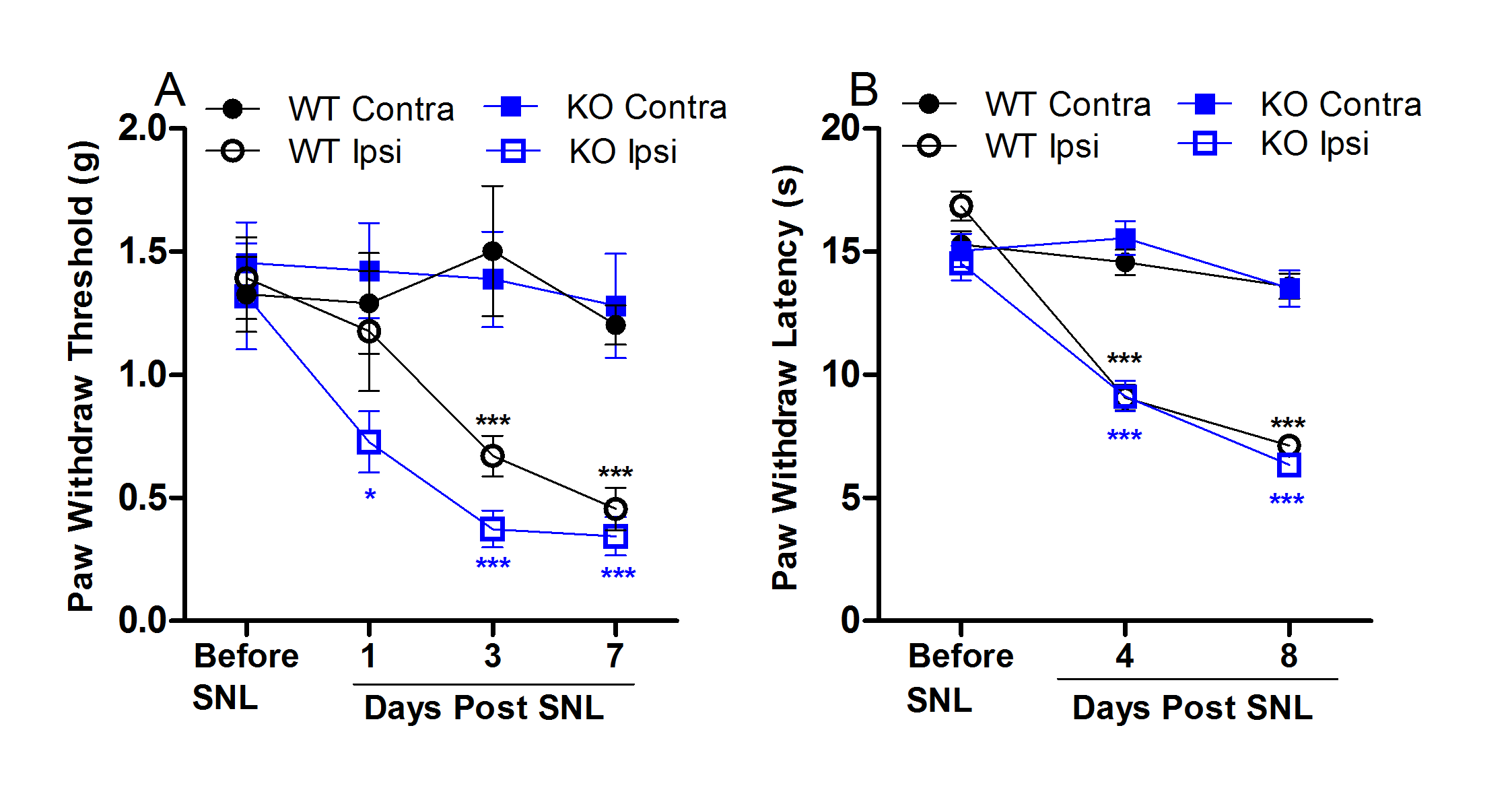

Supplement: S3 Fig — (A) Development of tactile allodynia of WT and D2KO mice assessed in the von Frey filaments assay (n = 10). Two way ANOVA revealed significant treatment effects (F3,36 = 11.99, P < 0.0001), time effect (F3,108 = 8.557, P < 0.0001) and treatment x time interaction (F9,108 = 2.451, P = 0.0140) followed by Bonferroni post hoc test: contralateral vs ipsilateral, *P < 0.05, *** P < 0.001. (B) Development of thermal hyperalgeisa of WT and D2KO mice assessed in the hot box assay (n = 10). Two way ANOVA revealed significant treatment effects (F3,36 = 56.34, P < 0.0001), time effect (F2,72 = 84.75, P < 0.0001) and treatment x time interaction (F6,72 = 16.90, P < 0.0001) followed by Bonferroni post hoc test: contralateral vs ipsilateral, *** P < 0.001. (TIF) [file pone.0162875.s003.tif]
